# Supplementary material for: Exclusions for resolving urban badger damage problems: outcomes and consequences
Source: PeerJ. 2016 Oct 13;4:e2579. doi: 10.7717/peerj.2579 (PMC5068413; doi:10.7717/peerj.2579)
Supplement: Supplemental Information 2 — Brief description of each study site. [file peerj-04-2579-s002.doc]

**Supplementary material. Case studies**

The majority of cases involved a main sett affecting a single property, but ten cases affected multiple properties. In this latter situation, close co-operation between home owners was very important. In the few cases where neighbours were uncooperative, this slowed progress of the exclusion work, made work more challenging, usually due to issues of access, and appeared to increase stress experienced by the licensee (pers. obs.). The high level of cooperation between neighbours during the exclusion of two highly active main setts was, in our opinion, pivotal to their success. The following are brief descriptions of each case studied. All location names and personal identities are withheld.

**1** - An eleven hole main sett covering four back gardens (present for at least twelve years) in the east of England was trapped resulting in the capture of nine badgers; two male adults, one breeding female, two male cubs and three female cubs. A partial exclusion began during autumn 2004 followed by partial excavation and badger proofing work, which continued for a month. Badgers were excluded from this garden, the perimeter of which was proofed, but animals retained access to parts of the sett via entrance holes in neighbouring gardens. A dispute over payment of exclusion costs between neighbours limited cooperation.

**2 -** Five badgers were trapped at a main sett in the east of England; three adults (one male and two females) and three cubs. Some of these animals also used the sett described in case 1 above. The licensed exclusion began in the autumn of 2005 and was successfully excluded and destroyed after the 21 day exclusion period. It was then proofed with chain link and badgers did not regain access.

**3 -** In summer 2004, a single badger excavated a hole next to an overgrown grave in a Midlands churchyard. Several graves were undermined and human bones were exposed on the spoil heap. An exclusion licence was granted and the badger was excluded from the hole following scrub clearance and fitting of a one-way gate. Several days before the exclusion the badger excavated two more holes, one at each of two nearby properties. One of these was excluded using a one-way gate and chain link under licence during the autumn. The badger moved to a culvert under a house (the only sett found) and was found dead four months later, caught in a snare at the entrance to the sett. Relevant authorities were informed.

**4 –** During spring to summer 2005 three adult females, an adult male, male cub and female cub were caught at a main sett in the east of England prior to exclusion work. During radio tracking we failed to identify alternative setts for these badgers. Exclusion work was not completed and the badgers remained within the sett.

**5 -** A three hole sett, known for thirty years, was present in a single east of England garden, but the tunnel system potentially spread under several gardens. During summer 2005 two adult females and two female cubs were trapped. The holes were gated under licence, but insufficient chain-link was laid. Badgers got back into the sett via an entrance hole that was not identified during the exclusion period. Two other setts were identified nearby during daytime radio tracking and both tagged adults used these. One of these was excluded under license and an attempt was made to exclude the other, but this failed. A further outlier sett was found nearby soon after.

**6 -** A seven hole outlier sett along a grass railway embankment in the midlands, one of many along a short distance of embankment, was subject to a licensed exclusion using one-way gates and chain link in the summer of 2006. Three adult badgers, one male, a breeding female and a non- breeding female were trapped and tagged. The badgers made use of five other setts within their range. The tunnels extended under a private garden in which the patio had started to collapse. After failing to exclude the badgers the number of gates used and chain link deployed was extended along the embankment, but this also failed. Consequently, excavation was undertaken successfully and the private garden was proofed.

**7 –** A highly active four-hole main sett covered several private gardens in the south east. Four badgers (adult male, two adult females and a cub) were caught during the autumn of 2006. Three other setts were identified within the badgers’ ranges during daytime radio tracking sessions. An exclusion attempt using one-way gates was unsuccessful so excavation was undertaken one month later. The garden perimeters were proofed against further interference from badgers.

**8&9** – A large private property in Greater London suffered the undermining of its outdoor swimming pool by a small but very active main sett from 2006 to 2008. An inactive two-hole annex sett was located at the other end of the private garden. Understorey vegetation was removed back to the bare earth, but mature trees were left in situ. All entrance holes were gated and chain link was laid over the surface of the sett and surrounding area, but its edge was not buried in a trench. Approximately 50% of the garden area was covered in chain link. There was at least one other weak point in the chain link near an entrance hole adjacent to the swimming pool. Despite the presence of a sett a short distance away, and which the resident badgers occasionally used, they exploited the weak point in the chain link, gaining re-entry to the focal sett, which was not excluded during this attempt. The property was subsequently sold and the new owner received a licence to attempt a further exclusion two years after the initial attempt. By this time the sett had become much larger and more active. During this attempt, all vegetation was removed from the garden and chain link was laid over the whole area and was fixed to one-way gates installed over every entrance hole. During this disturbance badgers used up to three setts within their range. This time, the sett was successfully excluded and the garden was reinstated, at a total cost of approximately £110,000.

**10** – This case involved a highly active main sett spread across four gardens in the south east during 2006. The problem centred on the undermining of a storm water drain and foul water sewer, but signs of subsidence of a patio and severe damage to lawns were also apparent. The licence applicant consulted with local homeowners and received the support and engagement of all those affected. The licensees engaged a professional consultant to manage the sett exclusion and landscape the gardens. In advance of the exclusion, all fences between affected properties were removed, all vegetation (excluding a few very substantial trees) was removed down to the soil surface, leaving bare earth across the whole site. A trench of approximately 1.5m depth was dug around the site and galvanised chain link was laid over the ground surface and fed down into the bottom of the trench, which was then filled with hardcore rubble. One-way gates were fitted to every sett entrance hole, and the chain link was fixed around these. Once excluded, badgers continued to gain entrance to the sett by clawing at staples that had been left on the gates until they opened. We observed this behaviour using trail cameras positioned at sett entrances, and recorded badgers within the sett even when the licensee believed it had been vacated. After removal of the staples, the sett was rapidly vacated, and badgers moved to two other nearby setts within their range.

**11** – The small outlier sett in this case, was undermining a driveway, which was subsiding, on a single property in the south east during 2006. The licensee intended to cover the ground with chain link and to fit one-way gates. However, before action was taken against the sett all detectable activity ceased. At the same time, we identified two collared badgers caught at this sett as having moved to an alternative sett. Consequently, the licensee simply covered the ground with chain link, without fitting one-way gates, and succeeded in keeping badgers out of the sett.

**12** – A moderately active but reasonably large (five entrance holes) outlier sett was causing damage to a garden in the north west, including subsidence of a garden wall during 2007. No vegetation clearance was undertaken, and no chain link was initially used, but one-way gates were fitted to each hole. Badgers gained re-entry to the sett by digging around the gates, so the licensee fitted chain link around and fixed to each gate, but only covering approximately 20% of the vulnerable area. Nevertheless, the exclusion was successful and badgers took up residence in a sett a few hundred metres away.

**13&14** - This active main sett undermined land immediately adjacent to a fire exit of a council-run property in the midlands. During 2007 an artificial sett was built on neighbouring land, although the diameter of pipes used to construct the tunnels was inadequate, and were positioned so that it was possible for rain water to collect within the sett. Some understorey vegetation was cleared, but many trees and some bushes remained. Chain link was laid over the surface of the sett and immediately surrounding area, but was not buried into a trench at its edge. One-way gates were fitted to all entrance holes, but badgers dug under the chain link and around the gates, and were not excluded during 2007. We saw no evidence of the artificial sett being used by badgers. In 2008, a new contractor was employed to build a fence around the sett. The fence was made of galvanised chain link, which was buried into the ground. A one-way gate was fitted into the fence. Badgers were evicted from the sett, which was subsequently destroyed.

**15** – This sett in the south east was under a substantial temporary school building, making sett surveying challenging. Vegetation removal was not necessary, and chain link was installed around the base of the building, with one-way gates fitted into it, producing an effect not too dissimilar to the fence described above. However, a single annex hole adjacent to the building was gated, without chain link surrounding it. Badgers were successfully evicted from the sett during 2007, and took up residence in a nearby sett.

**16** – This case involved a large, active main sett spread across three gardens in the east of England during 2007. The licensee’s home had been devalued due to the sett’s proximity to his house and the entrance holes heading in the direction of it. One of the affected neighbours was supportive of the licence application, but the other was not. This neighbour’s garden contained the majority of the sett, and the home owner routinely fed badgers in her garden and welcomed their presence. After much debate, this home owner agreed to the temporary exclusion of badgers from the sett. Vegetation was partially cleared to facilitate fitting of one-way gates, but no chain link was used. In the other two gardens vegetation was cleared and the sett was excavated. After successful exclusion of the badgers, during which time they moved off into one of three setts within their range, two of the gardens were proofed by destroying the portion of the sett on their land and infilling with concrete. The gates were removed from the third garden, and badgers were allowed to re-enter.

**17** – A sports pavilion was undermined by a two-hole outlier sett in Greater London. A further two entrance holes were present within a neighbouring garden, but it could not be determined whether they were linked to the former holes. Vegetation, except for mature trees, was removed from the area surrounding the former holes and chain link was laid on the ground, without burying the edge into a trench. One way gates were fitted over the two holes and fixed to the chain link. One of the holes in the garden was soft blocked, the other was inactive. After successful exclusion, the chain link was left in situ and covered over with earth.

**18** – This large, active main sett was excavated into a bank at the edge of a private garden in the south east. The sett was causing the bank to erode, approximately one third of the garden having slipped away by the time of our survey. A little-used annex sett was present approximately 50m along the bank. Problems arose due to the consultant requiring access to an uncooperative neighbour’s garden, which took some time to resolve. Vegetation except mature trees was removed and chain link was laid over the main sett and surrounding area, with one-way gates fitted over each hole and fixed to the chain link. The chain link was not buried in a trench and no action was taken against the annex sett. After successful exclusion of the badgers, they moved to one of two setts a few hundred metres away along the same road. The sett was then destroyed during landscaping work to reinstate the bank and garden.

**19** – This active main sett in the midlands was undermining a steep bank on the boundary of two gardens, and excavations under the upper garden had resulted in part of the lawn collapsing. Vegetation consisted of mature trees, which were left in situ. One-way gates were fitted to all entrance holes, and chain link was fitted around each gate, covering approximately 25% of the sett surface. Three other setts were present along the bank, and badgers took up residence in these after they were successfully excluded from the focal sett.

**20** – Two holes of this north west outlier sett opened on land belonging to a council run property with another in a private garden. The licensee gained agreement from the council to conduct a complete exclusion. Spoil was removed from outside all holes, but the sparse vegetation (a few bushes) was left in situ. Each hole was gated, and chain link was fitted to each gate, and around the surrounding area, but only protected approximately 20% of the vulnerable area of the garden. The badger (a mature female) was successfully excluded from the sett and took up residence in a nearby sett within her range. This sett had become more active during the current year than it had been before. It was believed by the local badger consultant that this female had been excluded during the previous year from an outlier sett undermining a temporary school building and within approximately 200m of this case.

**21** – The main problem caused by this midlands outlier sett was due to the volume of spoil making the garden unusable. A local badger enthusiast fitted one way gates to each of the two holes, but the door of these gates was manufactured from weld mesh, instead of the recommended plywood. No vegetation was removed, no chain link was fitted and the gates were not checked, and they soon became stuck open due to a build up of spoil. The exclusion failed.

**22** – This main sett was undermining graves in a west country church yard, making gravestones unstable, and had made unusable an amenity grassland area belonging to a private courtyard development. Only after an exclusion had been attempted, which involved partial removal of vegetation and laying of chain link over approximately 65% of the vulnerable area and fitting of one-way gates to all visible holes, was a single hole annex sett discovered in a neighbouring garden. It was believed by the licensee that this annex sett was the cause of the exclusion failing.

**23** – A large, active main sett in a large private garden in central England was causing a major A road to subside. The city council conducted the exclusion to protect the road. All vegetation except substantial mature trees was removed and chain link was laid over the entire area of the sett and some distance beyond, but it was not buried into a trench. All holes were gated. The badgers repeatedly found their way underneath the chain link and dug around the one-way gates to re-enter the sett. After several attempts to reinforce the chain link and gates, some of the badgers evacuated the sett. One took up residence in a nearby sett within its range, but another appeared to move into a neighbouring social group. This animal was radiotracked to a private garden approximately 500m from the focal sett. The home owner had reported seeing a badger wearing a collar and suffering open wounds to its fore quarters. On investigation, we observed the free-living animal to within 10m, and concluded that the injuries were bite wounds to the shoulders.

**24** – This large (30 hole), active main sett in the south had made a garden unusable since entrance holes were not only present within a bank of earth at the bottom of the garden, but were also present across approximately 50% of the lawn. Some vegetation was removed prior to exclusion, but mature bushes and trees were left in situ. All holes were gated, and chain link fixed to these covered approximately 80% of the vulnerable area. Despite having access to at least two outlier setts within their range, badgers penetrated the chain link, regaining entry to the sett, which was not successfully excluded within 31 days of the gates being closed.

**25** – In a north west housing development of approximately 10-20 years of age, a private garden had been constructed from turf laid over builder’s sand. An active, single hole outlier sett was undermining a patio, which was subsiding, and several tonnes of sand had been excavated by badgers, and deposited on the lawn. No vegetation was removed and no chain link was used, but a one-way gate was fitted over the entrance hole. Despite having access to another sett within its range, the badger re-gained entry to the focal sett by digging around the gate.

**26** – An earth bank between a private housing development and a derelict club house in the midlands contained an active outlier sett, which undermined a private garden. The property next to the affected one also appeared to have entrance holes to a disused annex sett heading under its garden, but it was not possible to ascertain whether any structural damage was being caused. The sett entrances were all on the club’s land, and the club agreed to allow the affected home owner to exclude the badgers. No vegetation was removed and no chain link was laid down, but by the time one-way gates had been installed over all entrance holes, the badgers had moved to a rural sett within their range. The exclusion was considered a success, and the sett was destroyed.

**27** – A moderately active outlier sett in a midlands suburban woodland was undermining a retaining wall to a school that stood at the top of a cliff. After two attempts we trapped a single adult male badger, which had access to at least three other setts within its sizable range. Understorey vegetation was removed but mature trees, which were thought to maintain the integrity of the cliff, were left in situ. All entrance holes were gated and chain link was laid over approximately 90% of the vulnerable area and fixed to the gates, but the edge was not buried into a trench. Nevertheless, the sett was successfully excluded by 21 days of the gates being installed. Gates were subsequently removed and replaced with chain link, which remains in place.

**28** – This moderately active outlier sett in the north east was undermining a garden shed and had made approximately 40% of the garden unusable due to the presence of entrance holes and spoil on the lawn and borders. A neighbouring garden contained two additional holes, but the involvement of this neighbour was not sought by the licensee. Consequently, the sett was partially excluded, with gates fitted to all holes in the focal garden. Chain link was fitted to and around the gates, covering approximately 25% of the vulnerable area. Little vegetation was present around the sett, and none was removed. Badgers were successfully excluded from the portion of the sett in the licensee’s garden within 31 days of gate closure. However, the licensee later contacted us stating that badgers had re-gained entry into the sett.

**29** – An active two-hole outlier sett in the south had made unusable approximately 40% of a private garden. Foraging by badgers in the lawned area had caused such significant damage that the home owner had laid artificial turf over the affected area in an attempt to prevent it. Vegetation removal was not necessary, and both holes were gated, with chain link fixed to these and over approximately 100% of the affected area. Badgers were successfully excluded within 21 days of the gates being installed, and tagged badgers took up residence in a main sett in a private garden within 100m of this case.

**30 –** Three small terraced properties owned by a housing trust in the north west experienced considerable damage to a large proportion of their back gardens from 9 active holes associated with a main sett and a two-hole annexe. An adult male and a breeding female were trapped and collared, although the female shed her collar prior to release. All vegetation was removed from the gardens, all entrances were gated and 60% of the associated sett area covered with chain link on the surface. The housing trust also had an artificial sett constructed prior to the exclusion on an area of land at the end of the row of terraces. Despite these efforts the badgers regained access to the sett through weak points in the chain link where they were able to squeeze underneath it. More chain link was added and after several weeks of failing to exclude the badgers, an electric fence incorporating one-way gates was erected around the perimeter of the sett. This was also breached. After three months and several unsuccessful attempts to exclude the badgers the housing trust was granted a licence to excavate the sett to its destruction. By this time the male’s collar had failed.

**31 –** An active 34 hole main sett in the north east on a slope adjoining a property, had enlarged subsequent to a successful exclusion at another sett close by. This led to the house owner becoming concerned that the structural integrity of his house was being weakened due to the sett undermining his property. A licence was granted to exclude the sett completely. However, the exclusion was not undertaken because the consultant was unsure on how to proceed and the quote (£17,000) for the exclusion works was considered excessive by the property owner.

**32** – An active 10-hole main sett covered three back gardens in a residential suburb in the west. Three animals were caught during late summer 2010; a female cub and two adult males. The two adults were fitted with GPS collars. The exclusion was undertaken by an ecological consultant with experience of badger exclusions. One-way gates were fitted and closed in the late summer. The area above the sett was cleared of vegetation, fences and a compost heap. Chain link was laid prior to gate closure. A tagged male breached the proofing on several occasions, and no alternative setts were identified within the badgers’ range. Badgers were not excluded from the sett by the end of this study.
